# Clinical correlates of a negative cerebrospinal fluid α-synuclein seed amplification assay result in Parkinson’s disease

**DOI:** 10.1038/s41531-026-01346-3

**Published:** 2026-04-14

**Authors:** Andrea Mastrangelo, Isabel Wurster, Alice Ticca, Stefanie Lerche, Corrado Zenesini, Benjamin Röben, Angela Mammana, Ann-Kathrin Hauser, Edoardo Ruggeri, Christian Deuschle, Erica Vittoriosi, Giuseppe Mario Bentivenga, Claudia Schulte, Sabina Capellari, Kathrin Brockmann, Piero Parchi

**Affiliations:** 1 Department of Biomedical and Neuromotor Sciences, University of Bologna, Bologna, Italy; 2 IRCCS, Istituto delle Scienze Neurologiche di Bologna, Bologna, Italy; 3 Department of Neurodegeneration, Center of Neurology, Hertie Institute for Clinical Brain Research, German Center for Neurodegenerative Diseases, University of Tübingen, Tübingen, Germany; 4 German Center for Neurodegenerative Diseases, University of Tübingen, Tübingen, Germany

**Keywords:** Diseases, Medical research, Neurology, Neuroscience

## Abstract

We investigated the basis and clinical correlates of a negative cerebrospinal fluid (CSF) alpha-synuclein (α-syn) seed amplification assay result in patients with a clinical diagnosis of sporadic or *GBA1*-associated PD formulated by movement disorder specialists. Out of 473 participants with a confirmed PD diagnosis at the last follow-up, 62 (13.1%) were α-syn negative. Among them, 3 out of 15 with available longitudinal CSF samples converted to α-syn positive. Alpha-syn negative participants had more severe axial motor impairment, lower odds of hyposmia, REM sleep behaviour disorder and constipation. There were no differences in motor and cognitive progression between groups. CSF neurofilament light chain values were not associated with α-syn status. Besides possible misdiagnosis, the results indicate that α-syn negative PD comprises a distinct patient subgroup, possibly associated with a low burden or absence of Lewy body pathology. The results support the use of CSF α-syn SAA in PD patient stratification.

## Introduction

The presence of cytoplasmic intraneuronal accumulation of misfolded alpha-synuclein (α-syn) in the form of Lewy Bodies (LB) and Lewy neurites is considered the pathologic hallmark of Parkinson’s disease (PD), the second most common neurodegenerative disease after Alzheimer’s disease^1,2^. Until recently, demonstrating LB pathology was only feasible in post-mortem tissue, making the clinical diagnosis imperfect, especially in early disease stages. However, the introduction of the α-syn seed amplification assay (SAA) has recently provided a pathology-specific biomarker for this prevalent disorder, enabling, for the first time, the in vivo detection of LB pathology^3,4^. Based on the robust performance demonstrated by the SAA using cerebrospinal fluid (CSF) or skin samples in independent clinical and pathological cohorts^3–10^, two proposals for biological research diagnostic criteria for PD have been put forward^11,12^. In line with what has already occurred for patients with Alzheimer’s disease, the application of this biologically based framework is expected to lead to an improved early diagnosis of PD and the selection of biomarker-defined at-risk cohorts of patients in a prodromal stage^13^.

In this scenario, the negative outcome of α-syn SAA in a small, though not negligible, group of patients with a clinical PD diagnosis across different cohorts, involving about 10–15% of participants^3,14–17^, raises significant questions that need to be addressed. Although some negative results are likely related to the known suboptimal accuracy of clinical diagnostic criteria, especially at early clinical stages, as also recently suggested by a study showing an increase in α-syn SAA sensitivity when considering only diagnoses formulated after a 10-year follow-up^16^, misdiagnoses likely account for only a minority of α-syn SAA-negative individuals with an established clinical diagnosis of PD.

As disease-modifying therapies targeting pathological α-syn are being developed for PD patients, determining the clinical features associated with the absence of LB pathology biomarkers is crucial.

Taking advantage of one of the largest cohorts of well-characterized participants with sporadic or *GBA1*-associated PD with available CSF samples, we aimed to assess the association between negative CSF α-syn SAA status and clinical outcomes and measures of clinical progression. Moreover, in a subset of participants with longitudinal samples, we evaluated the stability of the negative CSF α-syn SAA status over time. Finally, we tested a subgroup of α-syn negative participants using whole-exome sequencing to exclude rare genetic forms of PD not associated with LB pathology. To improve the accuracy of PD clinical diagnosis, we also replicated all analyses in a subgroup of participants who had been followed for at least 3 years.

## Results

### Cohort definition and baseline demographic features and α-syn SAA results

A total of 542 participants with an initial diagnosis of PD were evaluated for inclusion in the study. Screening for pathogenic variants in *GBA1*, *LRRK2*, *PRKN* and *PINK1* was performed in all cases, and 49 carriers of variants in *LRRK2*, *PRKN* and *PINK1* were excluded from the study. Additionally, four participants were excluded due to insufficient CSF volume, and two due to an inconclusive α-syn SAA result. Among the remaining 487 participants, 415 (85.2%) tested positive by α-syn SAA at baseline, while 72 (14.8%) did not show α-syn seeding activity at baseline.

A subset of 14 participants had a change in clinical diagnosis at follow-up visits. The change in diagnosis was formulated by a movement disorder specialist blinded to the α-syn SAA result. Notably, the number of changes was significantly higher among the α-syn negative participants than among the positive ones (10/72 vs. 4/415, *p* < 0.001). The most frequent alternative diagnoses were Progressive supranuclear palsy (*n* = 4), Multiple system atrophy-Parkinsonian type (*n* = 3) and Dementia with Lewy Bodies (DLB) (*n* = 3). Of note, all subjects later classified as DLB belonged to the positive α-syn group. Details on individuals with a changed clinical diagnosis, including their CSF α-syn status, are reported in Supplementary Table 1.

**Fig. 1:**
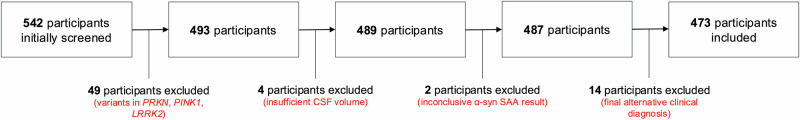
Study flow chart. α-syn alpha-synuclein, CSF cerebrospinal fluid, SAA seed amplification assay.

According to the inclusion criteria, the final cohort consisted of 473 PD patients (Fig. 1).

Demographic features of the included participants in the entire cohort are reported in Table 1. Median age at disease onset was 59 years. Females accounted for 32.5%. Pathogenic variants in *GBA1* were identified in 114 participants, accounting for 24.1% of the entire cohort.

**Table 1:** Demographic and basic clinical features of included participants

	Entire cohort (*N* = 473)	Extended follow-up subgroup (*n* = 320)
**Age at baseline**^**a**^	66.0 (57.0–72.0), 473	66.0 (57.0–71.0), 320
**Age at onset**^**a**^	59.0 (51.0–66.0), 473	58.0 (51.0–65.7), 320
**Females**	154/473 (32.5)	107/320 (33.4)
**Disease duration at baseline**^**a**^	5.1 (2.3–8.2), 473	5.3 (2.6–9.2), 320
**Time from first visit to last visit**^**a**^	5.2 (2.1–8.6), 473	7.7 (5.1–10.6), 320
**Time from baseline to last visit**^**a**^	3.0 (0.8–6.3), 473	4.6 (2.8–8.0), 320
***GBA1-*mutated**	114/473 (24.1)	86/320 (26.9)
**Abnormal DAT-SPECT**	107/107 (100)	69/69 (100)
***Clinical features at baseline***		
**H&Y stage**	2.0 (2.0–2.0), 471	2.0 (2.0–2.0), 319
**UPDRS part III**	24.0 (17.0–33.0), 428	24.0 (16.0–33.0), 298
**MoCA**	26.0 (23.0–28.0), 424	26.0 (24.0–28.0), 290
**Sniffin Sticks**	6.0 (4.0–8.0), 208	6.0 (4.0–8.0), 168
**BDI-2**	8.0 (4.0–13.0), 329	8.0 (4.0–13.0), 243
**LEDD**	460.0 (255.8–740.8), 466	471.0 (250.0–775.0), 318
**CSF NfL**	772.0 (558.3–1085.0), 399	747.0 (557.2–1059.0), 266
**CSF Aβ**_**42**_**/p-tau**_**181**_	19.3 (13.9–25.6), 417	18.7 (13.9–25.4), 282
***SAA results at baseline***		
**Positive**	411/473 (86.9)	282/320 (88.1)
**Negative**	62/473 (13.1)	38/320 (11.9)
***CSF samples available***		
**One**	331/473 (70.0)	194/320 (60.6)
**More than one**	142/473 (30.0)	126/320 (39.4)
**2**	83/142 (58.4)	72/126 (57.1)
**3**	23/142 (16.2)	18/126 (14.3)
**4**	15/142 (10.6)	15/126 (11.9)
**5**	7/142 (4.9)	7/126 (5.5)
> **5**	14/142 (9.8)	14/126 (11.1)

Continuous variables are expressed as median (interquartile range) and number of participants with available data. Dichotomous variables are expressed as number/number of participants with available data (%). Sniffin sticks are expressed as the number of the correctly identified sticks (out of 12). NfL values are expressed in pg/ml. Baseline was defined as the timepoint of the first available CSF sample.

*BDI* Beck Depression Inventory, *CSF* cerebrospinal fluid, *DAT-SPECT* dopamine transporter single-photon emission computed tomography, *H&Y* Hoehn and Yahr, *LEDD* Levodopa Equivalent Daily Dose, *MoCA* Montreal Cognitive Assessment, *NfL* neurofilament light chain protein, *SAA* seed amplification assay; *UPDRS* Unified Parkinson’s Disease Rating Scale.

^a^Expressed in years.

Similar demographic features characterized the subgroup with at least three years of follow-up (*n* = 320) (extended follow-up subgroup) (Table 1). Details on demographic features of sporadic PD participants are reported in Supplementary Table 2.

At baseline, 411 participants (86.9%) showed a positive α-syn SAA status (α-syn positive). Accordingly, a negative assay result was detected in 62 (13.1%) individuals (α-syn negative). The proportion of α-syn negative participants was slightly lower in the extended follow-up subgroup (38/320, 11.9%) (Table 1).

### Longitudinal stability of the negative α-syn status in participants with multiple samples available

For 142 participants (30.0%), two (83/142, 58.4%) or more longitudinal CSF samples were available (Table 1). Among them, 15 (10.6%) tested negative by α-syn SAA at baseline. However, three of these α-syn SAA-negative participants at baseline showed a positive α-syn SAA status at later samples (i.e., α-syn converters). Specifically, two of these participants had two CSF samples each and both converted to positive in the second sample. The third participant had three CSF samples available, with a negative result on the first and a positive result on the subsequent two.

Conversely, in the other 12 participants (80.0%), the negative α-syn SAA result remained stable over time (N of CSF samples available: median 2.0, IQR 2.0–3.0; time from first to last CSF sample: median 3.9 years, IQR 2.1–5.9 years). Details on α-syn converters and on those with longitudinal stable negative α-syn status are reported in Supplementary Table 3.

### Association of baseline negative α-syn status with baseline clinical outcomes

Baseline demographic features and clinical outcomes stratified by baseline CSF α-syn status are reported in Table 2 for the entire cohort and in Table 3 for the extended follow-up subgroup. Details on medication use among participants stratified by baseline CSF α-syn status are shown in Supplementary Table 4.

**Table 2:** Comparison of baseline clinical features between α-syn positive and α-syn negative participants in the entire cohort

	Entire cohort (*N* = 473)		
	α-syn positive (*n* = 411)	α-syn negative (*n* = 62)	*p* value
**Age at baseline**^**a**^	66.0 (57.0–71.0), 411	69.5 (59.7–76.0), 62	**0.017**^**b**^
**Age at onset**^**a**^	59.0 (51.0–65.0), 411	62.0 (56.0–71.2), 62	**0.004**^**b**^
**Females**	123/411 (29.9)	31/62 (50.0)	**0.002**^**c**^
**Disease duration at baseline**^**a**^	5.1 (2.4–8.3), 411	4.8 (1.8–7.0), 62	0.261^b^
**Time from first visit to last visit**^**a**^	5.2 (2.1–8.6), 411	4.4 (0.0–8.9), 62	0.315^b^
**Time from baseline to last visit**^**a**^	3.0 (0.9–6.2), 411	2.8 (0.0–6.8), 62	0.470^b^
***GBA1*-mutated**	105/411 (25.5)	9/62 (14.5)	0.078^c^
**Abnormal DAT-SPECT**	95/95 (100)	12/12 (100)	0.999
**H&Y stage**	2.0 (2.0–2.0), 409	2.0 (2.0–2.6), 62	**0.001**^**b**^
**UPDRS part III**	24.0 (17.0–33.0), 374	26.5 (16.7–34.0), 54	0.777^b^
**MoCA**	26.0 (23.0–28.0), 369	26.0 (22.0–28.0), 55	0.881^b^
**Sniffin Sticks**	5.0 (4.0–7.0), 185	10.0 (7.0–10.0), 23	**<0.001**^**b**^
**BDI-2**	7.0 (4.0–12.0), 287	9.0 (5.0–16.0), 42	**0.019**^**b**^
**LEDD**	482.5 (260.0–750.0), 406	445.0 (182.0–632.5), 60	0.083^b^
**CSF NfL**	766.0 (552.9–1049.0), 355	837.3 (598.6–1803.0), 44	0.061^b^
**CSF Aβ**_**42**_**/p-tau**_**181**_	19.3 (14.0–25.9), 366	18.4 (12.3–23.6), 51	0.508^b^
***Motor features***			
**Motor wearing-off**	29/313 (9.3)	4/40 (10.0)	0.778^c^
**Dyskinesias**	27/254 (10.6)	1/26 (3.8)	0.490^c^
**Repeated falls**	57/317 (18.0)	16/38 (42.1)	**0.001**^**c**^
**Resting tremor**	179/315 (56.8)	18/39 (46.1)	0.233^c^
***Non-motor features***			
**RBD**	150/348 (43.1)	13/52 (25.0)	**0.015**^**c**^
**Visual hallucinations**	84/383 (21.9)	7/54 (13.0)	0.153^c^
**Constipation**	194/396 (49.0)	18/57 (31.6)	**0.016**^**c**^
**Orthostatic hypotension**	152/383 (39.7)	20/56 (35.7)	0.661^c^
**Urinary urge**	227/391 (58.0)	37/58 (63.8)	0.475^c^

Continuous variables are expressed as median (interquartile range) and number of participants with available data. Dichotomous variables are expressed as number/number of participants with available data (%). Sniffin sticks are expressed as the number of the correctly identified sticks (out of 12). NfL values are expressed in pg/ml. Baseline was defined as the timepoint of the first available CSF sample. *P* values were not corrected for multiple comparisons.

*P* values of statistically significant comparisons are shown in bold.

*BDI* Beck Depression Inventory, *CSF* cerebrospinal fluid, *DAT-SPECT* dopamine transporter single-photon emission computed tomography, *H&Y* Hoehn and Yahr, *LEDD* Levodopa Equivalent Daily Dose, *MoCA* Montreal Cognitive Assessment, *RBD* REM sleep behaviour disorder, *NfL* neurofilament light chain protein, *UPDRS* Unified Parkinson’s Disease Rating Scale.

^a^Expressed in years.

^b^As derived by Mann-Whitney test.

^c^As derived by Fisher’s exact test.

**Table 3:** Comparison of clinical features at baseline between α-syn positive and α-syn negative in the extended follow-up subgroup

	Extended follow-up subgroup (*n* = 320)		
	α-syn positive (*n* = 282)	α-syn negative (*n* = 38)	*p* value
**Age at baseline**^**a**^	66.0 (57.0–71.0), 282	66.5 (60.2–74.2), 38	0.120^b^
**Age at onset**^**a**^	57.5 (50.7–65.0), 282	60.5 (54.0–67.2), 38	0.084^b^
**Females**	87/282 (30.8)	20/38 (52.6)	**0.010**^**c**^
**Disease duration at baseline**^**a**^	5.3 (2.8–9.2), 282	5.3 (2.2–8.1), 38	0.584^b^
**Time from first visit to last visit**^**a**^	7.4 (5.1–10.7), 282	8.5 (4.9–10.3), 38	0.449^b^
**Time from baseline to last visit**^**a**^	4.5 (2.6–7.9), 282	5.0 (3.1–8.2), 38	0.402^b^
***GBA1*-mutated**	79/282 (28.0)	7/38 (18.4)	0.246^c^
**Abnormal DAT-SPECT**	60/60 (100)	9/9 (100)	0.999
**H&Y stage**	2.0 (2.0–2.0), 281	2.0 (2.0–3.0), 38	**0.003**^**b**^
**UPDRS part III**	24.0 (17.0–33.0), 263	23.0 (15.0–32.0), 35	0.642^b^
**MoCA**	26.0 (23.0–28.0), 256	27.5 (25.0–29.0), 34	0.089^b^
**Sniffin Sticks**	6.0 (4.0–7.0), 153	10.0 (7.0–11.0), 15	**<0.001**^**b**^
**BDI-2**	7.0 (4.0–12.0), 214	9.0 (5.0–14.5), 29	0.128^b^
**LEDD**	496.5 (260.0–823.8), 280	450.0 (203.8–723.8), 38	0.298^b^
**CSF NfL**	747.5 (559.5–1048.0), 240	768.5 (553.3–1163.0), 26	0.621^b^
**CSF Aβ**_**42**_**/p-tau**_**181**_	18.8 (14.1–25.9), 250	15.8 (10.9–23.1), 32	0.108^b^
***Motor features***			
**Motor wearing-off**	23/239 (9.6)	3/30 (10.0)	>0.999^c^
**Dyskinesias**	22/191 (11.5)	1/20 (5.0)	0.704^c^
**Repeated falls**	38/233 (16.3)	10/25 (40.0)	**0.012**^**c**^
**Resting tremor**	138/237 (58.2)	15/28 (53.6)	0.688^c^
***Non-motor features***			
**RBD**	110/252 (43.6)	7/37 (18.9)	**0.004**^**c**^
**Visual hallucinations**	61/271 (22.5)	3/38 (7.9)	0.052^c^
**Constipation**	142/276 (51.4)	10/37 (27.0)	**0.008**^**c**^
**Orthostatic hypotension**	118/266 (44.4)	13/38 (34.2)	0.294^c^
**Urinary urge**	157/269 (58.4)	22/37 (59.4)	>0.999^c^

Continuous variables are expressed as median (interquartile range) and number of participants with available data. Dichotomous variables are expressed as number/number of participants with available data (%). Sniffin sticks are expressed as the number of the correctly identified sticks (out of 12). NfL values are expressed in pg/ml. Baseline was defined as the timepoint of the first available CSF sample. *P* values of statistically significant comparisons are shown in bold. *P* values were not corrected for multiple comparisons.

*BDI* Beck Depression Inventory, *CSF* cerebrospinal fluid, *DAT-SPECT* dopamine transporter single-photon emission computed tomography, *H&Y* Hoehn and Yahr, *LEDD* Levodopa Equivalent Daily Dose, *MoCA* Montreal Cognitive Assessment, *RBD* REM sleep behaviour disorder, *NfL* neurofilament light chain protein, *UPDRS* Unified Parkinson’s Disease Rating Scale.

^a^Expressed in years.

^b^As derived by Mann-Whitney test.

^c^As derived by Fisher’s exact test.

Alpha-syn negative participants were older at both baseline and disease onset in the entire cohort (age at baseline: *p* = 0.017; age at onset: *p* = 0.004), but not in the extended follow-up subgroup. Alpha-syn negative individuals were more frequently female than those with a positive α-syn status (entire cohort: *p* = 0.002; extended follow-up subgroup: *p* = 0.010). There were no significant differences between α-syn negative and α-syn positive participants in disease duration at baseline, follow-up duration (Tables 2 and 3), and use of medications, including dopamine agonists, antidepressants or other psychoactive drugs (Supplementary Table 4).

We assessed the independent association between baseline negative α-syn status and baseline clinical outcomes through multivariable logistic regression models, with age, sex, disease duration and *GBA1* status as covariates. Results are reported in Table 4.

**Table 4:** Independent associations of baseline α-syn status with baseline clinical outcomes

	Entire cohort (*N* = 473)				Extended follow-up subgroup (*n* = 320)			
	*n*	Odds ratio	*p* value	*q* value	n	Odds ratio	*p* value	*q* value
**H&Y stage**	471	2.26 (1.43–3.56)	<0.001	**0.004**	319	2.19 (1.24–3.85)	0.007	**0.024**
**UPDRS part III**	428	1.00 (0.97–1.03)	0.920	0.920	298	0.99 (0.96–1.02)	0.678	0.768
**MoCA**	424	1.01 (0.94–1.08)	0.743	0.789	290	1.55 (1.02–1.31)	0.027	0.066
**Sniffin Sticks**	208	2.03 (1.58–2.76)	<0.001	**<0.001**	168	2.25 (1.62–3.45)	<0.001	**<0.001**
**BDI-2**	329	1.06 (1.01–1.11)	0.014	**0.048**	243	1.05 (0.99–1.11)	0.078	0.166
**LEDD**	466	0.99 (0.99–1.01)	0.171	0.323	318	0.99 (0.99–1.01)	0.314	0.534
**CSF NfL**	399	1.00 (0.99–1.01)	0.076	0.164	266	1.00 (0.99–1.01)	0.410	0.634
**CSF Aβ**_**42**_**/p-tau**_**181**_	417	1.01 (0.98–1.05)	0.503	0.570	282	0.98 (0.93–1.03)	0.528	0.732
***Motor features***								
**Motor wearing-off**	353	1.57 (0.47–5.25)	0.466	0.566	269	1.46 (0.36–5.91)	0.594	0.732
**Dyskinesias**	280	0.39 (0.04–3.45)	0.401	0.524	211	0.55 (0.06–5.07)	0.603	0.732
**Repeated falls**	355	3.81 (1.73–8.42)	0.001	**0.005**	258	4.42 (1.64–11.91)	0.003	**0.017**
**Resting tremor**	354	0.65 (0.33–1.29)	0.222	0.377	265	0.90 (0.40–2.01)	0.795	0.795
***Non-motor features***								
**RBD**	400	0.43 (0.22–0.86)	0.017	**0.048**	289	0.29 (0.12–0.71)	0.007	**0.024**
**Visual hallucinations**	437	0.45 (0.19–1.09)	0.077	0.164	309	0.22 (0.06–0.78)	0.019	0.054
**Constipation**	453	0.43 (0.23–0.80)	0.007	**0.030**	313	0.29 (0.13–0.65)	0.003	**0.017**
**Orthostatic hypotension**	439	0.75 (0.41–1.37)	0.347	0.524	304	0.56 (0.26–1.18)	0.129	0.244
**Urinary urge**	449	1.29 (0.71–2.32)	0.401	0.524	306	1.13 (0.54–2.34)	0.747	0.794

Results are derived from multivariable logistic regression models, including age at baseline, sex, disease duration at baseline and genetic status as covariates. Odds ratios are expressed as main value (95% confidence interval). *Q* values were derived from correction for multiple testing using False Discovery Rate at *α* = 0.05 through Benjamini-Hochberg; two independent corrections were performed (one for the entire cohort, one for the extended follow-up subgroup, 17 outcomes each). *Q* values of statistically significant associations are shown in bold.

*BDI* Beck Depression Inventory, *CSF* cerebrospinal fluid, *H&Y* Hoehn and Yahr, *LEDD* Levodopa Equivalent daily dose, *MoCA* Montreal Cognitive Assessment, *RBD* REM sleep behaviour disorder.

**Fig. 2:**
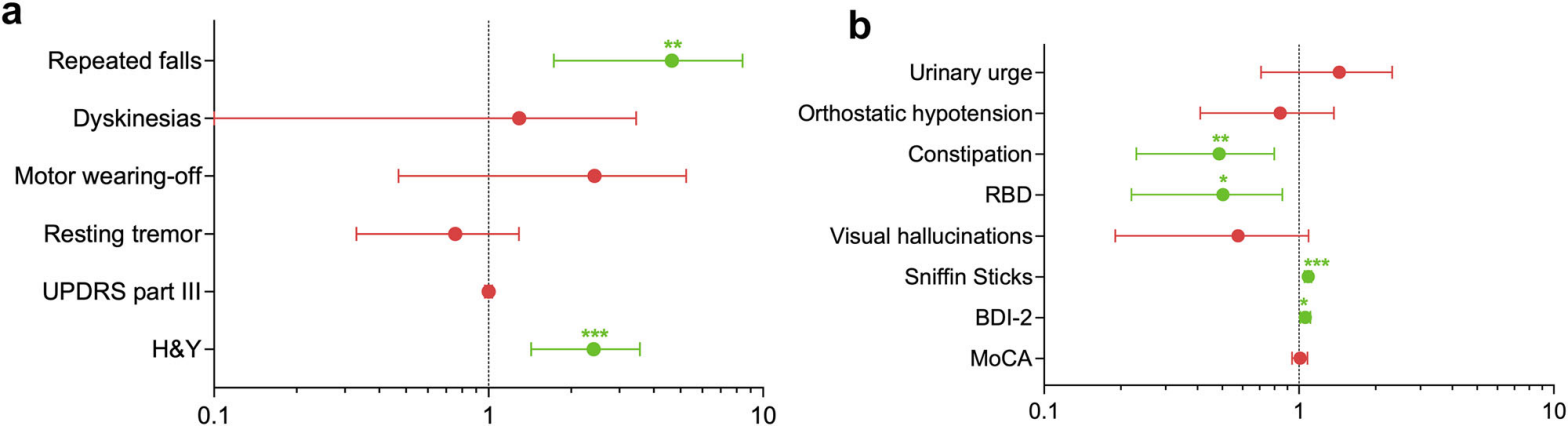
Independent associations between CSF α-syn negative status and baseline clinical outcomes. **a** Associations between α-syn negative status and baseline motor outcomes in the entire cohort. **b** Associations between α-syn negative status and baseline non-motor outcomes in the entire cohort. Odds ratios (OR) with relative 95% confidence intervals are displayed. OR values are derived from multivariable logistic regression models with binarized α-syn status as dependent variable and clinical outcomes as independent variable; all models are corrected for age at baseline, sex, disease duration at baseline and genetic status. Asterisks mark the statistically significant associations. H&Y Hoehn and Yahr stage, MoCA Montreal Cognitive Assessment, RBD REM sleep behaviour disorder, UPDRS Unified Parkinson’s Disease Rating Scale.

In both the entire cohort and the extended follow-up subgroup, a negative α-syn status was independently associated with a more severe motor impairment, as shown by higher Hoehn and Yahr (H&Y) stage (entire cohort: *p* < 0.001, *q* = 0.004; extended follow-up subgroup: *p* = 0.007, *q* = 0.024) and higher odds of repeated falls (entire cohort: *p* = 0.001, *q* = 0.005; extended follow-up subgroup: *p* = 0.003, *q* = 0.017) (Table 4 and Fig. 2). In contrast, α-syn status was not significantly related to the presence of resting tremor at baseline (entire cohort: *p* = 0.222; extended follow-up subgroup: *p* = 0.795).

Alpha-syn negative participants had higher Sniffin Sticks scores, indicating a better olfactory performance (*p* and *q* < 0.001 in both the entire cohort and extended follow-up subgroup) (Table 4 and Fig. 2).

Moreover, in both groups, a negative α-syn status was also independently associated with lower odds of REM sleep behaviour disorder (RBD) (entire cohort: *p* = 0.017, *q* = 0.048; extended follow-up subgroup: *p* = 0.007, *q* = 0.024) and constipation (entire cohort: *p* = 0.007, *q* = 0.030; extended follow-up subgroup: *p* = 0.003, *q* = 0.017). In the entire cohort but not in the extended follow-up subgroup, a negative α-syn status was associated with higher scores on the Beck Depression Inventory-2 (BDI-2) scale for depression, suggesting greater impairment (*p* = 0.014, *q* = 0.048) (Table 4, Fig. 2). In the extended follow-up subgroup, α-syn negative participants were less cognitively impaired, as suggested by higher Montreal Cognitive Assessment (MoCA) scores (*p* = 0.027) and had lower frequency of visual hallucinations (*p* = 0.019), but the results did not survive correction for multiple testing (*q* values 0.066 and 0.054, respectively).

Baseline α-syn status was unrelated to baseline CSF neurofilament light chain (NfL) levels in both the entire cohort (*p* =  0.076) and the extended follow-up subgroup (*p* = 0.410). Notably, we also found no association between CSF NfL and α-syn status in the subgroup of participants with higher motor axial impairment (i.e., H&Y ≥ 3 or repeated falls at baseline) (Supplementary Table 5).

Lastly, CSF Aβ_42_/p-tau_181_ ratio was unrelated to α-syn status in both the entire cohort (*p* = 0.503) and the extended follow-up subgroup (*p* = 0.528).

Distribution of baseline demographic and clinical features by baseline CSF α-syn status in the subgroup of sporadic PD is reported in Supplementary Table 6. Secondary multivariable logistic regression models in this subgroup showed largely overlapping results. As an exception, a baseline negative α-syn status was associated with higher CSF NfL values (Supplementary Table 7).

### Association of a negative α-syn status with measures of disease progression and longitudinal outcomes

To assess the association between negative α-syn status and the longitudinal development of disease milestones, we performed multivariable time-dependent Cox regression analyses, with α-syn status varying over time, and age, sex, disease duration and *GBA1* status as covariates. Results are reported in Table 5.

**Table 5:** Independent associations of time-varying α-syn status with measures of disease progression

	Entire cohort (*N* = 473)				Extended follow-up subgroup (*n* = 320)			
	*n*	Hazard ratio	*p* value	*q* value	*n*	Hazard ratio	*p* value	*q* value
***Motor milestones***								
**Postural instability**	321	0.53 (0.26–1.06)	0.074	0.105	254	0.54 (0.27–1.10)	0.090	0.147
**Motor wearing-off**	280	0.30 (0.12–0.74)	0.009	0.072	230	0.30 (0.12–0.74)	0.009	0.072
**Dyskinesias**	222	0.27 (0.08–0.89)	0.031	0.083	178	0.27 (0.08–0.91)	0.034	0.115
**Repeated falls**	240	1.04 (0.51–2.13)	0.904	0.904	193	0.86 (0.39–1.90)	0.719	0.810
***Non-motor milestones***								
**RBD**	193	0.40 (0.18–0.92)	0.031	0.083	162	0.43 (0.19–0.97)	0.043	0.115
**Visual hallucinations**	290	0.44 (0.19–1.04)	0.062	0.105	232	0.48 (0.20–1.13)	0.092	0.147
**Orthostatic hypotension**	210	0.87 (0.43–1.78)	0.709	0.810	161	0.91 (0.45–1.88)	0.810	0.810
**Severe cognitive impairment**	318	0.38 (0.13–1.12)	0.079	0.105	262	0.41 (0.14–1.22)	0.110	0.147

Results are derived from multivariable time-varying Cox regression models with α-syn status varying over time. Age at CSF sampling, sex, disease duration at CSF sampling and genetic status were considered as covariates. Hazard ratios are expressed as main value (95% confidence interval). Postural instability was defined as a H&Y stage ≥ 3; severe cognitive impairment was defined as a MoCA score ≤ 18. *Q* values were derived from correction for multiple testing using False Discovery Rate at *α* = 0.05 through Benjamini-Hochberg; two independent corrections were performed (one for the entire cohort, one for the extended follow-up subgroup, eight outcomes each).

*H&Y* Hoehn and Yahr, *MoCA* Montreal Cognitive Assessment, *RBD* REM sleep behaviour disorder.

**Fig. 3:**
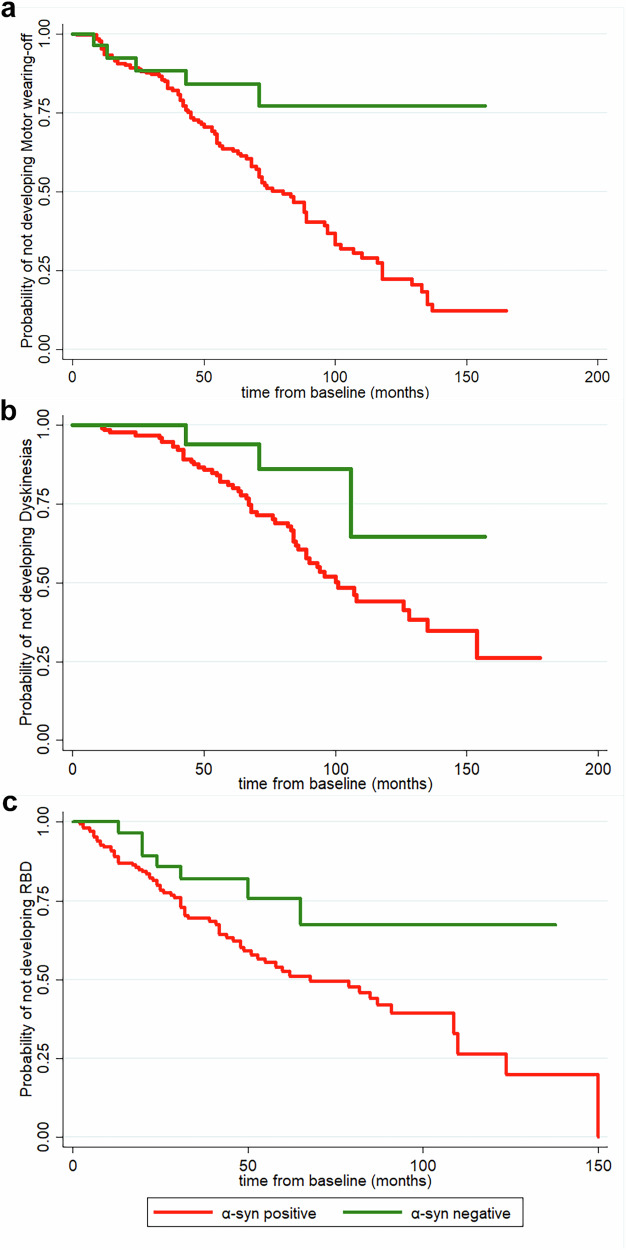
Associations between α-syn status and development of disease milestones. Kaplan-Meier curves showing the associations between baseline α-syn status and the risk of developing motor wearing-off (**a**) dyskinesias (**b**) and RBD (**c**) in the entire cohort. Alpha-syn negative participants are displayed in green, α-syn positive in red. Time is expressed in months. RBD REM sleep behaviour disorder.

In both the entire cohort and in the extended follow-up subgroup, a negative α-syn status was associated with a lower risk developing motor wearing-off (entire cohort: *p* = 0.009; extended follow-up subgroup: *p* = 0.009) and dyskinesias (entire cohort: *p* = 0.031; extended follow-up subgroup: *p* = 0.034). Moreover, the lack of CSF α-syn seeding activity was associated with a lower risk of developing RBD at follow-up (entire cohort: *p* = 0.031; extended follow-up subgroup: *p* = 0.043) (Fig. 3). However, the statistical significance of these associations was lost after correction for multiple testing (Table 5).

CSF α-syn status did not significantly affect the risk of developing postural instability, repeated falls, visual hallucinations, OH or severe cognitive impairment (Table 5).

Overlapping results were obtained in the sporadic PD subgroup (Supplementary Table 8).

Scores at Unified Parkinson’s Disease Rating Scale (UPDRS) part III and MoCA, as well as H&Y stage values, significantly worsened over time in both the entire cohort (H&Y: *β* = 0.0003, 95%CI 0.0003–0.0005, *p* < 0.001; UPDRS part III: *β* = 0.004, 95%CI 0.003–0.006, *p* < 0.001; MoCA: *β* = −0.0009, 95%CI −0.001 to −0.0005, *p* < 0.001) and the extended follow-up subgroup (H&Y: *β* = 0.0004, 95%CI 0.0003–0.0005, *p* < 0.001; UPDRS part III: *β* = 0.004, 95%CI 0.003–0.006, *p* < 0.001; MoCA: *β* = −0.001, 95% CI −0.001 to −0.0005, *p* < 0.001), suggesting longitudinal motor and cognitive deterioration. Similarly, Levodopa Equivalent Daily Dose (LEDD) values increased over time in both groups (entire cohort: *β* = 0.28, 95%CI 0.12–0.44, *p* < 0.001; extended follow-up subgroup: *β* = 0.25, 95%CI 0.08–0.42, *p* = 0.004) (Supplementary Table 9). However, α-syn status did not affect the rates of progression in either the entire cohort (Likelihood ratio tests: H&Y *p* = 0.568; UPDRS part III *p* = 0.951; MoCA *p* = 0.607; LEDD *p* = 0.934) or the extended follow-up subgroup (Likelihood ratio tests: H&Y *p* = 0.553; UPDRS part III *p* = 0.941; MoCA *p* = 0.329; LEDD *p* = 0.885) (Supplementary Table 9).

The secondary analysis in the subgroup of sporadic PD participants showed largely overlapping results (Supplementary Table 10).

### Genetic findings in α-syn SAA-negative PD participants

We performed whole-exome sequencing (WES) analysis in a subset of α-syn negative participants with disease onset <50 years (7 out of 8). Demographic and basic clinical features of α-syn negative participants with available WES data are reported in Supplementary Table 11. For the majority of these (6/7, 85.7%), only one CSF sample was available. A single participant had two CSF samples, both of which tested negative for α-syn SAA.

Overall, three variants were detected in two different participants (Supplementary Table 12). However, none of the variants detected was considered clearly causative of the PD phenotype.

Specifically, in a participant (#1), a heterozygous variant in *SLC6A3* (c.911 G > A; p.R304Q) and a heterozygous variant in *SYNJ1* (c.4286 G > A; p.R1429Q) were identified. The former was classified as likely benign by Varsome and as a Variant of Uncertain Significance (VUS) by Franklin Genoox and ClinVar, while the latter was recognized as a VUS by all tools. In another participant, a heterozygous variant in *DCTN1* (c.3731 A > G; p.Y1244C) was found; this was classified as likely benign by Varsome and as VUS by Franklin Genoox and ClinVar.

For five participants, no variants were detected in the analysed genes.

## Discussion

We report a detailed description of clinical features and disease trajectories in patients with a clinical diagnosis of PD formulated by Movement Disorder specialists who tested negative for CSF α-syn SAA.

We found that, at baseline, a negative CSF α-syn status was associated with more severe axial motor impairment, a lower frequency of non-motor manifestations, including hyposmia, RBD, and constipation and a higher BDI-2 score, suggesting a higher prevalence of depression. In the longitudinal analysis, α-syn SAA-negative participants had a lower risk of developing RBD and complications of dopaminergic therapy, including motor wearing-off and dyskinesias, although the findings did not retain statistical significance after multiple-testing correction. Conversely, motor and cognitive progression rates did not differ between the two groups.

Hyposmia has already been reported as a correlate of α-syn SAA positivity in clinical cohorts of patients with PD^14^. It is also a well-known correlate of LB pathology from neuropathological studies^18,19^. RBD is also strongly associated with α-syn pathology^20^, and constipation is included among the prodromal symptoms of PD^21,22^.

The finding of α-syn SAA-negative individuals among patients with a clinical diagnosis of PD raises critical questions about the basis of nigro-striatal dysfunction in these patients, with implications for the potential utility of CSF α-syn SAA and the application of the neuronal synuclein disease framework to clinical trial design. Several hypotheses can be formulated to explain the absence of α-syn seeding activity. The first one to be considered is that at least some α-syn negative participants may represent PD misdiagnosis. Neuropathological studies indicate that the clinical diagnosis of PD is not highly accurate, particularly at initial assessments and when performed outside specialised centres^23,24^. The introduction of the MDS criteria^25^ may have improved diagnostic performance, especially at later disease stages^26^. Still, in a recent neuropathological study, only a slight improvement in overall diagnostic accuracy was observed after their introduction^24^. In our cohort, the higher proportion of females among the α-syn negative participants, compared with the male predominance usually reported in PD^27,28^, may suggest enrichment for other conditions lacking a sex-specific distribution. Similarly, the independent association between α-syn negativity and more severe motor axial impairment, suggested by both higher H&Y stage and higher frequency of falls, may indicate the presence of atypical parkinsonism (AP) or vascular parkinsonism in a subset of cases. However, α-syn positive and α-syn negative participants did not show overall differences in motor progression over time, as measured by both the time to reach motor disease milestones and the longitudinal trajectories of H&Y stage and UPDRS part III scores. This may suggest that the proportion of AP within the α-syn negative subgroup is small. Consistently, LEDD escalation over time did not differ between the two groups, who have similar motor progression rates, arguing against major differences in dopaminergic responsiveness. Moreover, all significant associations observed in the entire cohort were replicated in the extended follow-up subgroup, where diagnostic accuracy is expected to be higher. Additionally, resting tremor, a hallmark of PD, was not differentially distributed between the two groups. Furthermore, the baseline and follow-up disease durations did not differ between groups and are inconsistent with the natural history of AP. Finally, baseline CSF NfL levels, an excellent discriminator between PD and AP^29–31^, were not independently associated with α-syn status in either the entire cohort or the extended follow-up subgroup, further supporting a low rate of AP misdiagnosis. As the only exception, we found significantly higher NfL values in the sporadic PD subgroup (i.e., after excluding *GBA1*-mutations carriers), suggesting a possible slightly higher misdiagnosis rate in this subcohort.

A second hypothesis is that some PD patients may test negative for CSF α-syn due to a low brain LB burden at the time of assessment. Recent studies highlighted that the burden and stage of the underlying LB pathology affect CSF α-syn SAA sensitivity^5–8^. However, incomplete sensitivity primarily involves individuals with LB pathology at Braak stage 3 or lower, who are usually considered affected by incidental LB pathology. Conversely, patients with PD are expected to harbour LB pathology at Braak stage > 3 at clinical diagnosis. Moreover, as the disease progresses, the LB load is expected to increase, thereby further enhancing α-syn SAA sensitivity. In our cohort, only 3 of 15 α-syn negative participants with longitudinal CSF samples available (20%) converted to positive α-syn status. This suggests that a low LB burden may explain a negative CSF α-syn status in a few PD cases. In these subjects, a greater vulnerability of nigral cells to α-syn deposition and toxicity, or a different pattern of LB pathology distribution with less involvement of structures involved in sleep, autonomic regulation, and olfactory function, can be hypothesised. Indeed, α-syn positive participants exhibited a higher frequency of prodromal symptoms, such as hyposmia, constipation and RBD, consistent with more widespread LB pathology at the time of assessment, possibly extending to peripheral autonomic structures. Although the contribution of peripheral α-syn deposition to CSF α-syn SAA sensitivity remains currently unexplored, a higher LB load in the brainstem has been reported in subjects with peripheral involvement^32^. In line with this, a recent study demonstrated a higher CSF α-syn SAA sensitivity in PD patients with abnormal MIBG myocardial scintigraphy^15^, suggesting postganglionic sympathetic denervation and peripheral α-syn deposition. Future studies should compare CSF α-syn SAA performance with that of peripheral tissue-based assays, including skin and olfactory mucosa, across different PD phenotypes.

A third hypothesis is that a minority of patients with a PD phenotype may truly lack LB pathology. This has been clearly documented in carriers of pathogenic variants in the *PINK1* and *PRKN* genes and, to a lesser extent, the *LRRK2* gene^14,17,33–35^, who were accordingly excluded from our cohort. Subjects with a clinical diagnosis of PD lacking specific neuropathological features besides nigral atrophy have been reported, albeit rarely^36–40^. As a main limitation, these studies did not screen for the most frequently associated PD genes. To explore this possibility, we performed WES in a subset of early-onset α-syn negative participants who have the highest probability of a monogenic disease. However, we did not identify any pathogenic variants that could explain the clinical phenotype, as all detected variants were either of uncertain significance or heterozygous, whereas the disease requires biallelic disruption.

In this context, the presence of underlying alternative pathologies should also be considered as a possible explanation for some α-syn negative participants. As an example, recent studies demonstrated the presence of tau nigral pathology in subjects with motor deficits, possibly driving the striatal dopaminergic dysfunction^41^. Moreover, in a recent report, lower Αβ_42_ levels were found in a small group of α-syn negative PD participants as compared to the positive ones, suggesting a higher prevalence of underlying AD pathology^42^. In a larger cohort, we did not confirm these findings, showing that CSF Αβ_42_/p-tau_181_ levels were unrelated to CSF α-syn status.

Finally, although not supported by robust evidence, a distinct conformational strain may underlie some cases of PD and exhibit different biological behaviour, including lower detectability by the currently available SAA. In this view, these subjects may show a distinct clinical phenotype, characterized by a more severe motor and axial impairment, a lower rate of non-motor manifestations and complications related to dopaminergic therapy, as well as a higher frequency of depression and female predominance. Interestingly, a recent meta-analysis indicated that depression in PD is associated with female sex as well as with a higher motor and axial impairment^43^. Although highly speculative, this hypothesis warrants further biochemical and neuropathological investigation.

Along this line, a recent study demonstrated the presence of pathological α-syn oligomers in the brains of *LRRK2*-PD patients who lacked LB pathology at neuropathological examination^44^. At present, it is not known whether these oligomeric species have seeding activity and reach the CSF. Nevertheless, the possibility that α-syn negative PD participants harbour α-syn pathology associated with a predominant oligomeric form of aggregation cannot be entirely excluded.

The lack of neuropathological confirmation is the main limitation of our study. Although our α-syn SAA has been validated across independent neuropathological cohorts with very high sensitivity and specificity^3,8^, few participants had a clinical diagnosis of PD. Additionally, diagnoses in our cohort were not based on the most recent MDS criteria^25^, possibly reducing accuracy. However, all participants were evaluated by movement disorder specialists at a referral centre and, in most cases, longitudinally followed, mitigating this limitation^45^. Replication of our findings in the extended follow-up subgroup further strengthens diagnostic reliability. As a further limitation, our α-syn SAA cannot detect α-syn seeds of multiple system atrophy patients, unlike alternative assays^46^. We cannot, therefore, exclude that some of our α-syn negative participants actually harbour a synucleinopathy different from LB disease.

Additionally, we recognize that the percentage of *GBA1*-mutated PD participants in our cohort was higher than that reported in previous studies^14^. This likely reflects the fact that the University Hospital of Tübingen is.a specialized referral Center for genetic PD. Importantly, our results were largely replicated in the subgroup of sporadic PD patients.

Moreover, WES analysis was available only for a small subset of α-syn negative participants, limiting the generalizability of the results. Nonetheless, the probability of a monogenic disorder is likely lower in subjects with an older disease onset. Furthermore, we acknowledge the limitations of WES in identifying genetic abnormalities other than exonic variants, such as intronic variants, repeat expansions or other structural variations, which can also be associated with a parkinsonian syndrome.

Finally, RBD assessment relied on PD-NMS questionnaire items rather than polysomnography, possibly reducing diagnostic precision.

Conversely, the inclusion of well-characterised participants from one of the largest PD clinical cohorts with available CSF samples is the main strength of our work. Additionally, screening all participants for variants in the most common PD-associated genes, as well as the very high accuracy of our α-syn SAA assay, are significant strengths of our study.

In conclusion, in a large cohort of patients diagnosed with PD, a negative CSF α-syn status is associated with female sex, higher baseline motor impairment, as well as with lower frequency of non-motor manifestations, such as hyposmia, constipation and RBD. However, in the longitudinal analysis, α-syn negative and positive participants showed a comparable risk of developing disease milestones and overlapping disease trajectories.

## Methods

### Inclusion and exclusion criteria

We studied subjects with a clinical diagnosis of PD who were initially evaluated at the University Hospital of Tübingen (Germany) outpatient clinic or ward between 2002 and 2024 and who had at least one CSF sample available. Inclusion criteria for the study were as follows: 1) a clinical diagnosis of PD at the last available visit; 2) sufficient CSF volume to perform α-syn SAA; and 3) a conclusive SAA result (see below). Subjects carrying heterozygous or bi-allelic mutations in *LRRK2*, *PRKN*, and *PINK1* (*n* = 49) were excluded. The final entire cohort comprised 473 participants. The study flow-chart is summarised in Fig. 1.

### Clinical assessment

Clinical diagnoses of PD were attributed according to the UK Brain Bank Society Criteria^47^. All participants underwent a baseline neurological assessment at the time of the first CSF sampling, including a comprehensive neurological examination and an anamnestic interview. Because the first CSF collection did not occur at the first visit for each participant, most participants of the whole cohort (*n* = 304/473, 64.3%) had one or more assessments before the CSF baseline visit. From baseline, most participants of the whole cohort (*n* = 364/473, 76.9%) were longitudinally followed for a median time of 4.0 years (interquartile range (IQR) 2.1–7.5) and reassessed periodically (in most cases, yearly).

For each participant, the following quantitative measures/scores at clinical scales were obtained at baseline (time of the first available CSF sample) and at each follow-up visit: UPDRS part III (for participants evaluated between 2002 and 2008, UPDRS part III old version; for other participants, MDS-UPDRS part III), MoCA (for participants evaluated before 2009, Mini-Mental State Examination (MMSE) scores were converted into MoCA equivalents)^48^, BDI-2 and LEDD. Disease stage was categorized by the modified H&Y Scale. LEDD was calculated as previously described^49,50^. Olfactory function was assessed using the Sniffin’ sticks test (Screening 12 test) and expressed as the number of correctly identified sticks. The presence of the following motor clinical features was assessed at baseline and at each subsequent visit through neurological examination/interview: dyskinesias, motor wearing-off and repeated falls. Motor examination was performed in the medication-ON state. Resting tremor at baseline was defined by a score≥1 on at least one of the resting tremor items in UPDRS part III. The following non-motor features were assessed at each visit through interview/PD-NMS questionnaire^51^: RBD, constipation, orthostatic hypotension, urinary urge and visual hallucinations. Orthostatic hypotension was defined as a decrease by at least 20 mmHg in systolic pressure (or by 10 mmHg in diastolic pressure) within three minutes of standing^52^.

### CSF biomarker analyses

SAA analyses were performed at the Neuropathology Laboratory of the Institute of Neurological Sciences in Bologna (NP-Lab), blinded to clinical status, as previously described^3,53^, with minor modifications. CSF samples were shipped to the NP-Lab in three separate batches: the first in 2021, the second in 2024, and the last in 2025. Samples from the first (n = 340) and the third (n = 10) shipment were initially tested in quadruplicates. Samples were classified as positive if they had at least two positive replicates. Samples showing unclear results (1/4) were retested twice, and a final positive result was attributed to those showing at least four out of 12 positive replicates. Samples from the second shipment (*n* = 418) were initially assessed by α-syn endpoint dilution quantitative SAA^54^ and were therefore tested in octuplicates. These were considered positive if at least 3 of 8 replicates were positive. Unclear samples (1/8 or 2/8) were retested in quadruplicate three times, and the final binary classification was based on the three additional runs: samples with ≥4/12 positive replicates were considered positive. For all shipments, samples were considered negative if they yielded 0/4 or 0/8 positive replicates at first screening, or <4/12 positive replicates after retesting without showing a 2/4 result in any of the three runs. Samples with <4/12 positive replicates, but one 2/4 result, were considered inconclusive for the present work and excluded from analyses.

CSF Aβ_42_, p-tau_181_ and NfL levels were available in a subset of participants at baseline. Analyses were performed by board-certified laboratory technicians who were blinded to the clinical data. CSF levels of Aβ_42_ and p-tau_181_ were measured using ELISA kits from INNOTEST, Fujirebio GmbH, Germany. Intra-assay coefficients of variation were below 15%. The Αβ_42_/p-tau_181_ ratio was considered as supportive of underlying Alzheimer’s disease (AD) pathology^55^. CSF NfL levels were determined using the UmanDiagnostics, NF-light® assay, an enzymatic two-site immunoassay for quantitative NfL determinations in human CSF. Two internal longitudinal quality control samples were run on each plate. Intra-assay coefficients of variation were below 15%.

### Genetic analyses

Genetic screening for pathogenic variants in the genes *GBA1*, *LRRK2*, *PRKN*, and *PINK1* was performed at the University Hospital of Tübingen, as previously described^17^.

In a subset of participants with a negative CSF α-syn SAA result, disease onset <50 years (7 out of 8), and an available DNA sample, WES was performed at the NP-Lab as described^56^. Briefly, the sample library was prepared using xGenTM DNA EZ Library Prep Kit (IDT), enriched with xGen Exome Research Panel v2 (IDT) probes, and then sequenced with 2 × 100 bp paired-end reads on a NovaSeq 6000 instrument (Illumina). Sequencing was performed with an average coverage of 131.7X, and the coverage of 20X was over 99% of all samples. Bioinformatic analysis followed the GATK v.4.2.0.0 workflow for germline variant discovery, aligning to reference genome GRCh38/hg38. We prioritised rare variants with autosomal inheritance in genes associated with dystonia/parkinsonism (DYT/PARK) and parkinsonism (PARK) according to the Movement Disorder Society Genetic Mutation Database (MDSgene) as of October 2025^57^. Variants of interest were classified according to the American College of Medical Genetics guidelines^58^, and the evaluation of clinical consequences was based on ClinVar^59^, Franklin Genoox^60^ and Varsome databases^61^.

### Statistical analyses

Statistical analyses were performed using GraphPad Prism V. 7 (GraphPad, La Jolla, CA, USA) and Stata SE v.14.2 (StataCorp, College Station, TX, USA). To maximise the diagnostic accuracy of the clinical diagnosis of PD, all statistical analyses were performed on the entire cohort and on a subgroup of participants who were clinically followed for at least 3 years from the first visit (extended follow-up subgroup). Secondary analyses were conducted in the subgroup of sporadic PD participants.

Normality of continuous variables was assessed using the Shapiro-Wilk and Kolmogorov-Smirnov tests. As variables were not normally distributed, comparisons were made using the Mann-Whitney test. Fisher’s exact test was employed for categorical variables.

The association between baseline α-syn status and baseline clinical outcomes (either scores on clinical scales, presence/absence of motor/non-motor features, or CSF biomarkers values) was assessed using multivariable logistic regression models with binarized α-syn status as the dependent variable and clinical outcomes as independent variables. Age at baseline, sex, disease duration at baseline and genetic status (sporadic PD or *GBA1*-PD) were considered as covariates in all models. Results are expressed as OR and 95%CI. Results of multivariable logistic regression analyses underwent correction for multiple testing using the false discovery rate (FDR) method at *α* = 0.05 according to Benjamini-Hochberg. Independent corrections were performed for the entire cohort, the extended follow-up subgroup and the sporadic PD subgroup (17 outcomes each).

The association between time-varying α-syn status and the risk of disease milestones was evaluated using multivariable time-dependent Cox regression models. The time of entry into the analysis was the date of CSF sampling, while the time of endpoint was the date of milestone achievement (i.e., the date of the follow-up visit in which the presence of the specific milestone was firstly documented) or the last follow-up visit (for censored participants). Subjects who already achieved the milestone at baseline were excluded. Age at baseline, sex, disease duration at baseline and genetic status were considered as covariates in all models. The following disease milestones were considered: postural instability (as defined by a H&Y stage ≥ 3), motor wearing-off, dyskinesias, repeated falls, RBD, visual hallucinations, OH, and severe cognitive impairment (as determined by a MoCA score≤18), largely overlapping with previous published works^62,63^. Results are expressed as HR and 95%CI. The Kaplan-Meier curve was used to represent event probabilities over time. P values were corrected through FDR at *α* = 0.05 according to Benjamini-Hochberg. Three independent corrections were performed (see above, eight outcomes each).

Finally, we assessed the longitudinal variation in quantitative measures of disease severity (H&Y stage, UPDRS part III score, MoCA score, LEDD) from baseline to last available follow-up visit using linear mixed-effects models with random intercepts and slopes. To evaluate whether the slope of change over time differed by CSF α-syn status, an interaction term between time and α-syn status was included. Results are expressed as β coefficients and 95%CI. The significance of the interaction was assessed using a likelihood ratio test comparing models with and without the interaction term. Subjects with a negative CSF α-syn status at baseline and later converting to a positive test were deemed as α-syn positive for this analysis. *P* values of the likelihood test were corrected for multiple testing as described above (four outcomes each). All analyses were two-sided.

### Ethics approval and consent to participate

The study was conducted in compliance with the revised Declaration of Helsinki for the protection of human participants and Good Clinical Practice guidelines and was approved by the Ethics Committee of the University of Tübingen (26/2007BO1, 404/2010BO1, 199/2011BO1, 702/2013BO1). All participants gave written informed consent.

## Supplementary information

Supplementary Information

## Data Availability

The datasets generated and/or analyzed during the current study are not publicly available due to privacy and ethical concerns, but are available from the corresponding authors (piero-parchi@unibo.it or kathrin.brockmann@uni-tuebingen.de) upon reasonable request.
