# Supplementary material for: Clinical correlates of a negative cerebrospinal fluid α-synuclein seed amplification assay result in Parkinson’s disease
Source: NPJ Parkinsons Dis. 2026 Apr 14;12:97. doi: 10.1038/s41531-026-01346-3 (PMC13086934; doi:10.1038/s41531-026-01346-3)
Supplement: Supplementary file 1 — Supplementary Information [file 41531_2026_1346_MOESM1_ESM.pdf]

Supplementary Table 1
Demographic and clinical features of participants changing clinical diagnosis during follow-up

	Last clinical diagnosis	Sex	Age at onset	Age at CSF sampling	N of CSF samples	CSF SAA result	Years from first visit to last visit	Years from baseline to last visit
**#1**	Vascular PK	F	74	83	1	N	2.0	1.0
**#2**	MSA-P	F	61	65	1	N	2.0	0.0
**#3**	PSP	M	68	72	1	N	2.8	0.0
**#4**	ET	F	67	72,74	2	N,N	11.6	7.5
**#5**	PSP	M	61	65,66,68.70	4	N,N,N,N	5.2	4.4
**#6**	DLB	M	61	63	1	P	3.0	2.0
**#7**	DLB	M	66	70	1	P	2.0	2.0
**#8**	MSA-P	M	52	55.57	2	N,N	4.7	2.8
**#9**	PSP	M	77	79	1	N	3.4	3.4
**#10**	DLB	M	58	62	1	P	3.0	0.0
**#11**	MSA-P	M	51	52,53	2	N,N	1.0	0.0
**#12**	CBD	M	79	83	1	P	1.0	1.0
**#13**	PSP	M	69	75	1	N	5.0	0.0
**#14**	Leukodystrophy	F	62	67	1	N	3.0	3.0

Baseline was defined as the timepoint of the first available CSF sample. Abbreviations: CBD, Corticobasal Degeneration; CSF, cerebrospinal fluid; DLB, Dementia with Lewy Body; ET, Essential Tremor; F, female; M, male; MSA-P, Multiple System Atrophy-Parkinsonian type; N, negative; P, positive; PK, Parkinsonism; PSP, Progressive Supranuclear Palsy; SAA, seed amplification assay.

Supplementary Table 2
Demographic and basic clinical features of included participants in the sporadic PD subgroup

	**Sporadic PD subgroup (n=359)**
**Age at baseline[a]**	67.0 (58.0–72.0), 359
**Age at onset[a]**	60.0 (53.0–67.0), 359
**Females**	113/359 (31.5)
**Disease duration at baseline[a]**	4.8 (2.3–7.8), 359
**Time from first visit to last visit[a]**	4.6 (1.9–8.6), 359
**Time from baseline to last visit[a]**	3.0 (0.8–6.3), 359
**Abnormal DAT-SPECT**	82/82 (100)
Clinical features at baseline	
**H&Y stage**	2.0 (2.0–2.0), 357
**UPDRS part III**	23.0 (16.0–32.0), 321
**MoCA**	26.0 (23.0–28.0), 316
**Sniffin Sticks**	6.0 (4.0–8.0), 144
**BDI-2**	7.0 (4.0–13.0), 236
**LEDD**	450.0 (250.0–712.0), 355
**CSF NfL**	789.0 (584.4–1085.0), 299
**CSF $A\beta_{42}$/p-tau_{181}**	19.1 (13.6–26.2), 316
SAA results at baseline	
**Positive**	306/359 (85.2)
**Negative**	53/359 (14.8)
CSF samples available	
**One**	259/359 (72.1)
**More than one**	100/359 (27.8)
**2**	64/100 (64.0)
**3**	13/100 (13.0)
**4**	9/100 (9.0)
**5**	5/100 (5.0)
**>5**	9/100 (9.0)

[a]Expressed in years

Continuous variables are expressed as median (interquartile range) and number of participants with available data. Dichotomous variables are expressed as number/number of participants with available data (%). Sniffin sticks are expressed as the number of the correctly identified sticks (out of 12). NfL values are expressed in pg/ml. Baseline was defined as the timepoint of the first available CSF sample. Abbreviations: BDI, Beck Depression Inventory; CSF, cerebrospinal fluid; H&Y, Hoehn and Yahr; LEDD, Levodopa Equivalent Daily Dose; MoCA, Montreal Cognitive Assessment; NfL, neurofilament light chain protein; PD, Parkinson’s disease; DAT-SPECT, dopamine transporter single-photon emission computed tomography; UPDRS, Unified Parkinson’s Disease Rating Scale.

Supplementary Table 3
Demographic and clinical features of α-syn negative participants with longitudinal CSF samples available

	Status	Sex	Age at onset	Age at baseline	N of CSF samples	Disease duration at baseline (years)	H&Y at baseline	UPDRS part III at baseline	MoCA at baseline
**#1**	α-syn C	M	61	66	3	4.8	2.5	34	28
**#2**	α-syn C	F	42	44	2	1.5	2	11	30
**#3**	α-syn C	M	51	61	2	9.9	3	34	25
**#4**	α-syn PN	F	54	61	2	6.8	2	16	28
**#5**	α-syn PN	M	67	79	2	12.8	3	18	NA
**#6**	α-syn PN	M	74	77	3	2.7	2	11	29
**#7**	α-syn PN	M	41	52	2	11.6	2	10	24
**#8**	α-syn PN	F	57	63	3	6.8	2	17	29
**#9**	α-syn PN	F	64	71	2	7.0	2.5	19	29
**#10**	α-syn PN	F	74	79	3	4.3	2	23	27
**#11**	α-syn PN	M	67	67	4	0.8	1	14	25
**#12**	α-syn PN	F	76	78	2	2.3	2	13	29
**#13**	α-syn PN	M	57	58	5	0.8	2	7	27
**#14**	α-syn PN	F	64	66	2	1.8	2	34	28
**#15**	α-syn PN	F	57	58	2	1.8	1	6	24

Baseline was defined as the timepoint of the first available CSF sample. Abbreviations: α-syn, alpha-synuclein; α-syn C; alpha-synuclein converters; α-syn PN, alpha-synuclein persistently negative; CSF, cerebrospinal fluid; F, female; H&Y, Hoehn and Yahr stage; M, male; MoCA, Montreal Cognitive Assessment; NA, not available; UPDRS, Unified Parkinson's Disease Rating Scale.

Supplementary Table 4
Distribution of selected medications stratified by baseline α-syn status in the entire cohort and the extended follow-up subgroup

	Entire cohort (N=473)			Extended follow-up subgroup (n=320)		
**At baseline**						
	**α-syn positive (n=411)**	**α-syn negative (n=62)**	**p value[a]**	**α-syn positive (n=282)**	**α-syn negative (n=38)**	**p value[a]**
**Dopamine agonists**	239/407 (58.7)	27/57 (47.4)	0.117	185/281 (65.8)	22/37 (59.5)	0.466
**Antidepressants**	82/407 (20.1)	12/57 (21.0)	0.861	59/281 (21.0)	7/37 (18.9)	0.999
**Antipsychotics**	37/407 (9.1)	6/57 (10.5)	0.634	20/281 (7.1)	5/37 (13.5)	0.190
**Benzodiazepines**	7/407 (1.7)	1/57 (1.7)	0.999	7/281 (2.5)	1/37 (2.7)	0.999
**Entire clinical history**						
	**α-syn positive (n=411)**	**α-syn negative (n=62)**	**p value[a]**	**α-syn positive (n=282)**	**α-syn negative (n=38)**	**p value[a]**
**Dopamine agonists**	298/407 (73.2)	36/57 (63.2)	0.118	230/281 (81.8)	30/37 (81.1)	0.999
**Antidepressants**	126/407 (31.0)	19/57 (33.3)	0.761	96/281 (34.2)	13/37 (35.1)	0.999
**Antipsychotics**	91/407 (22.4)	10/57 (17.5)	0.494	69/281 (24.6)	8/37 (21.6)	0.839
**Benzodiazepines**	21/407 (5.2)	1/57 (1.7)	0.500	21/281 (7.5)	1/37 (2.7)	0.490

[a]As derived by Fisher's exact test

Dichotomous variables are expressed as number/number of participants with available data (%). Baseline was defined as the timepoint of the first available CSF sample. Entire clinical history here refers to the time from baseline to last available clinical follow-up. P values were not corrected for multiple comparisons.

Supplementary Table 5
Association of baseline α-syn status with NfL values in the subgroup of participants with motor axial impairment

	Entire cohort (n=399)					
	H&Y≥3 at baseline (n=64)			Repeated falls at baseline (n=56)		
	α-syn + (n=50) median (IQR)	α-syn - (n=14) median (IQR)	p value[a]	α-syn + (n=43) median (IQR)	α-syn - (n=13) median (IQR)	p value[a]
**CSF NfL**	1052 (725–1458)	1342 (879–1924)	0.168	997 (603–1367)	1060 (510–1961)	0.420
	Odds ratio		p value[b]	Odds ratio		p value[b]
**CSF NfL**	1.00 (0.99–1.00)		0.753	1.00 (0.99–1.00)		0.357
	Extended follow-up subgroup (n=266)					
	H&Y≥3 at baseline (n=40)			Repeated falls at baseline (n=39)		
	α-syn + (n=31) median (IQR)	α-syn - (n=9) median (IQR)	p value[a]	α-syn + (n=31) median (IQR)	α-syn - (n=8) median (IQR)	p value[a]
**CSF NfL**	997 (744–1371)	1060 (581–1613)	0.999	1048 (603–1341)	746 (431–1739)	0.505
	Odds ratio		p value[b]	Odds ratio		p value[b]
**CSF NfL**	0.99 (0.99–1.00)		0.957	1.00 (0.99–1-00)		0.244

[a]As derived by Mann-Whitney test
[b]As derived by multivariable logistic regression models. The following covariates were considered: age at baseline, sex, disease duration at baseline, genetic status.
Odds ratios are reported as main value (95% confidence interval). NfL values are expressed in pg/ml. In all groups, only participants with available CSF NfL values are included. P values were not corrected for multiple comparisons. Abbreviations: CSF, cerebrospinal fluid; H&Y, Hoehn and Yahr; IQR, interquartile range; NfL, neurofilament light chain.

Supplementary Table 6
Comparison of baseline clinical features between α-syn positive and α-syn negative participants in the subgroup of sporadic PD

	**Sporadic PD subgroup (n=359)**		
	**α-syn positive (n=306)**	**α-syn negative (n=53)**	**p value**
**Age at baseline**[a]	66.0 (58.0–72.0), 306	69.0 (59.5–75.5), 53	0.136[b]
**Age at onset**[a]	60.0 (52.0–66.2), 306	61.0 (55.0–70.5), 53	0.106[b]
**Females**	88/306 (28.7)	26/53 (49.0)	**0.006**[c]
**Disease duration at baseline**[a]	4.6 (2.3–7.8), 306	5.0 (2.2–7.5), 53	0.959[b]
**Time from first visit to last visit**[a]	5.1 (2.0–8.6), 306	3.8 (0.0–8.7), 53	0.110[b]
**Time from baseline to last visit**[a]	3.1 (0.9–6.4), 306	2.3 (0.0–5.0), 53	0.135[b]
**Abnormal DAT-SPECT**	72/72 (100)	10/10 (100)	0.999
**H&Y stage**	2.0 (2.0–2.0), 304	2.0 (2.0–3.0), 53	**<0.001**[b]
**UPDRS part III**	23.0 (16.0–31.0), 276	27.0 (18.0–34.0), 45	0.328[b]
**MoCA**	26.0 (23.0–28.0), 270	25.5 (21.7–28.0), 46	0.594[b]
**Sniffin Sticks**	5.0 (4.0–8.0), 127	9.0 (7.5–10.0), 17	**<0.001**[b]
**BDI-2**	7.0 (3.0–12.0), 203	13.0 (6.0–16.0), 33	**0.002**[b]
**LEDD**	455.5 (260.0–727.3), 304	450.0 (176.0–640.0), 51	0.248[b]
**CSF NfL**	783.8 (569.9–1020.0), 264	1122.0 (662.7–1910.0), 35	**0.008**[b]
**CSF $A\beta_{42}$/p-tau_{181}**	19.1 (13.5–26.8), 274	19.0 (13.6–24.6), 42	0.979[b]
Motor features			
**Motor wearing-off**	14/235 (5.9)	3/32 (9.4)	0.439[c]
**Dyskinesias**	17/185 (9.2)	1/18 (5.5)	0.999[c]
**Repeated falls**	39/235 (16.6)	12/30 (40.0)	**0.005**[c]
**Resting tremor**	130/226 (57.5)	14/31 (45.2)	0.247[c]
Non-motor features			
**RBD**	110/267 (41.2)	10/44 (22.7)	**0.020**[c]
**Visual hallucinations**	60/289 (20.8)	7/45 (15.5)	0.549[c]
**Constipation**	143/299 (47.8)	16/48 (33.3)	0.085[c]
**Orthostatic hypotension**	112/291 (38.5)	14/47 (29.8)	0.329[c]
**Urinary urge**	172/298 (57.7)	32/49 (65.3)	0.350[c]

[a]Expressed in years
[b]As derived by Mann-Whitney test
[c]As derived by Fisher's exact test
Continuous variables are expressed as median (interquartile range) and number of participants with available data. Dichotomous variables are expressed as number/number of participants with available data (%). Sniffin sticks are expressed as the number of the correctly identified sticks (out of 12). NfL values are expressed in pg/ml. Baseline was defined as the timepoint of the first available CSF sample. P values were not corrected for multiple comparisons. Abbreviations: BDI, Beck Depression Inventory; CSF, cerebrospinal fluid; H&Y, Hoehn and Yahr; LEDD, Levodopa Equivalent Daily Dose; MoCA, Montreal Cognitive Assessment; NfL, neurofilament light chain protein; PD, Parkinson's disease; RBD, REM sleep behaviour disorder; DAT-SPECT, dopamine transporter single-photon emission computed tomography; UPDRS, Unified Parkinson's Disease Rating Scale.

Supplementary Table 7
Independent associations of baseline α-syn status with baseline clinical outcomes in the sporadic PD subgroup

	Sporadic PD subgroup (n=359)			
	**n**	**Odds ratio**	**p value**	**q value**
**H&Y stage**	357	2.56 (1.54–4.37)	<0.001	**0.003**
**UPDRS part III**	321	1.01 (0.98–1.04)	0.606	0.687
**MoCA**	316	0.98 (0.91–1.06)	0.670	0.712
**Sniffin Sticks**	144	2.26 (1.63–3.47)	<0.001	**<0.001**
**BDI-2**	236	1.07 (1.02–1.13)	0.009	**0.042**
**LEDD**	355	0.99 (0.99–1.01)	0.224	0.394
**CSF NfL**	299	1.00 (1.00–1.01)	0.016	**0.045**
**CSF $A\beta_{42}$/p-tau_{181}**	316	1.02 (0.98–1.06)	0.255	0.394
Motor features				
**Motor wearing-off**	267	2.01 (0.40–7.59)	0.336	0.408
**Dyskinesias**	203	0.82 (0.04–5.59)	0.863	0.863
**Repeated falls**	265	3.27 (1.31–8.10)	0.010	**0.042**
**Resting tremor**	257	0.64 (0.29–1.38)	0.253	0.394
Non-motor features				
**RBD**	311	0.38 (0.17–0.80)	0.015	**0.045**
**Visual hallucinations**	334	0.62 (0.23–1.45)	0.299	0.408
**Constipation**	347	0.48 (0.24–0.91)	0.029	0.070
**Orthostatic hypotension**	338	0.60 (0.29–1.17)	0.146	0.310
**Urinary urge**	347	1.40 (0.74–2.74)	0.336	0.408

Results are derived from multivariable logistic regression models, including age at baseline, sex, disease duration at baseline as covariates. P values of statistically significant associations are shown in bold. Odds ratios are expressed as main value (95% confidence interval). Postural instability was defined as a H&Y stage≥3; severe cognitive impairment was defined as a MoCA score≤18. Q values were derived from correction for multiple testing using False Discovery Rate at α=0.05 through Benjamini-Hochberg (a single correction was performed, 17 outcomes). Abbreviations: BDI, Beck Depression Inventory; CSF, cerebrospinal fluid; H&Y, Hoehn and Yahr; LEDD, Levodopa Equivalent daily dose; MoCA, Montreal cognitive assessment; RBD, REM sleep behaviour disorder; UPDRS, Unified Parkinson's Disease Rating Scale.

Supplementary Table 8
Independent associations of time-varying α-syn status with measures of disease progression in the sporadic PD subgroup

	**Sporadic PD subgroup (n=359)**			
	**n**	**Hazard ratio**	**p value**	**q value**
Motor milestones				
**Postural instability**	250	0.54 (0.23–1.27)	0.160	0.320
**Motor wearing-off**	216	0.38 (0.14–1.04)	0.061	0.203
**Dyskinesias**	160	0.64 (0.19–2.15)	0.474	0.542
**Repeated falls**	184	1.41 (0.61–3.29)	0.419	0.542
Non motor milestones				
**RBD**	155	0.27 (0.09–0.79)	0.017	0.136
**Visual hallucinations**	222	0.39 (0.14–1.10)	0.076	0.203
**Orthostatic hypotension**	167	0.79 (0.35–1.78)	0.570	0.570
**Severe cognitive impairment**	239	0.55 (0.15–2.00)	0.361	0.542

Results are derived from multivariable time-varying Cox regression models with α-syn status varying over time. Age at CSF sampling, sex and disease duration were considered as covariates. P values of statistically significant associations are shown in bold. Hazard ratios are expressed as main value (95% confidence interval). Postural instability was defined as a H&Y stage≥3; severe cognitive impairment was defined as a MoCA score≤18. Q values were derived from correction for multiple testing using False Discovery Rate at α=0.05 through Benjamini-Hochberg (a single correction was performed, eight outcomes). Abbreviations: H&Y, Hoehn and Yahr; MoCA, Montreal Cognitive Assessment; PD, Parkinson's disease; RBD, REM sleep behaviour disorder.

Supplementary Table 9
Effects of α-syn status on longitudinal variation of quantitative measures of disease severity

Entire cohort (n=364)				
	**β coefficient**	**p value[a]**	**Interaction between time and CSF α-syn status[b]**	
			**p value**	**q value**
**H&Y (n=364)**	0.0004 (0.0003 – 0.0005)	**<0.001**	0.568	0.951
**UPDRS part III (n=336)**	0.004 (0.003 – 0.006)	**<0.001**	0.951	0.951
**MoCA (n=323)**	-0.0009 (-0.001 – -0.0005)	**<0.001**	0.600	0.951
**LEDD (n=360)**	0.28 (0.12 – 0.44)	**<0.001**	0.934	0.951
**Extended follow-up subgroup (n=290)**				
	**β coefficient**	**p value[a]**	**Interaction between time and CSF α-syn status[b]**	
			**p value**	**q value**
**H&Y (n=290)**	0.0004 (0.0003 – 0.0005)	**<0.001**	0.553	0.941
**UPDRS part III (n=273)**	0.004 (0.003 – 0.006)	**<0.001**	0.941	0.941
**MoCA (n=267)**	-0.001 (-0.001 – 0.0005)	**<0.001**	0.329	0.941
**LEDD (n=289)**	0.25 (0.08 – 0.42)	**0.004**	0.885	0.941

[a]Derived from linear mixed-effects models evaluating the interaction between clinical outcomes and time
[b]Derived from the p value of the likelihood ratio test comparing linear mixed-effects models with and without the interaction term between time and α-syn status.
In both the entire cohort and in the extended follow-up subgroup, only participants with at least one clinical assessment after baseline are included. Discrepancies in the number of subjects included for each clinical outcome are related to possible missing values. Q values were derived from correction for multiple testing using False Discovery Rate at α=0.05 through Benjamini-Hochberg; two independent corrections were performed (one for the entire cohort, one for the extended follow-up subgroup, four outcomes each). Time is expressed in months. Abbreviations: H&Y, Hoehn and Yahr stage; LEDD, Levodopa Equivalent Daily dose; MoCA, Montreal Cognitive Assessment; UPDRS, Unified Parkinson’s Disease Rating Scale.

Supplementary Table 10
Effects of α-syn status on longitudinal variation of quantitative measures of disease severity in the sporadic PD subgroup

Sporadic PD subgroup (n=276)				
	**β coefficient**	**p value[a]**	**Interaction between time and CSF α-syn status[b]**	
			**p value**	**q value**
**H&Y (n=276)**	0.0004 (0.0003 – 0.0005)	**<0.001**	0.558	0.689
**UPDRS part III (n=252)**	0.004 (0.002 – 0.006)	**<0.001**	0.689	0.689
**MoCA (n=239)**	-0.0008 (-0.001 – -0.0003)	**0.001**	0.568	0.689
**LEDD (n=272)**	0.28 (0.23 – 0.33)	**<0.001**	0.649	0.689

[a]Derived from linear mixed-effects models evaluating the interaction between clinical outcomes and time
[b]Derived from the p value of the likelihood ratio test comparing linear mixed-effects models with and without the interaction term between time and α-syn status.
Only participants with at least one clinical assessment after baseline are included. Discrepancies in the number of subjects included for each clinical outcome are related to possible missing values. Q values were derived from correction for multiple testing using False Discovery Rate at α=0.05 through Benjamini-Hochberg (a single correction was performed, four outcomes). Time is expressed in months.
Abbreviations: H&Y, Hoehn and Yahr stage; LEDD, Levodopa Equivalent Daily dose; MoCA, Montreal Cognitive Assessment; PD, Parkinson's disease, UPDRS, Unified Parkinson's Disease Rating Scale.

Supplementary Table 11
Demographic and clinical features of α-syn negative participants with available whole sequencing examination

	Sex	Age at onset	Age at baseline	N of CSF samples	Disease duration at baseline (years)	H&Y at baseline	UPDRS part III at baseline	MoCA at baseline
**#1**	M	27	35	1	8.9	2	35	NA
**#2**	M	33	46	1	13.8	3	29	NA
**#3**	M	41	52	2	11.6	2	10	24
**#4**	M	44	50	1	6.3	2	29	26
**#5**	M	49	52	1	3.0	2	15	30
**#6**	M	37	39	1	2.5	2	NA	28
**#7**	M	38	42	1	5.0	1	8	23

Baseline was defined as the timepoint of the first available CSF sample. Abbreviations: CSF, cerebrospinal fluid; F, female; H&Y, Hoehn and Yahr stage; M, male; MoCA, Montreal Cognitive Assessment; NA, not available; UPDRS, Unified Parkinson's Disease Rating Scale.

Supplementary Table 12
Results of whole exome sequencing on selected PD participants with a negative α-syn status

	Gene	rsID	Variant	Varsome	Franklin	Clinvar	Clinical phenotype according to MDSgene
#1	No variants detected						
#2	*SLC6A3*	rs748308614	c.911G>A (p.R304Q) Het	Lik Ben (BP4, BP1, PM2)	VUS (PM2)	VUS	Dystonia-parkinsonism
	SYNJ1	rs112469776	c.4286G>A (p.R1429Q) Het	VUS (PM2, BP1)	VUS (PM2, BP4)	VUS	Atypical parkinsonism
#3	No variants detected						
#4	*DCTN1*	rs752422008	c.3731A>G (p.Y1244C) Het	Lik Ben (BP4)	VUS (PM2)	VUS	Atypical parkinsonism
#5	No variants detected						
#6	No variants detected						
#7	No variants detected						

Abbreviations: Ben, Benign; Het, Heterozygous; Hom, Homozygous; Lik Ben, Likely Benign; Lik Pat, Likely Pathogenic; NA, not available; Pat, Pathogenic; VUS, variant of uncertain significance
